# Feasibility of Biochar Derived from Sewage Sludge to Promote Sustainable Agriculture and Mitigate GHG Emissions—A Review

**DOI:** 10.3390/ijerph191912983

**Published:** 2022-10-10

**Authors:** Mohammad Ghorbani, Petr Konvalina, Anna Walkiewicz, Reinhard W. Neugschwandtner, Marek Kopecký, Kazem Zamanian, Wei-Hsin Chen, Daniel Bucur

**Affiliations:** 1 Department of Agroecosystems, Faculty of Agriculture and Technology, University of South Bohemia in Ceske Budejovice, Branišovská 1645/31A, 370 05 Ceske Budejovice, Czech Republic; 2 Department of Natural Environment Biogeochemistry, Institute of Agrophysics, Polish Academy of Sciences, Doświadczalna 4, 20-290 Lublin, Poland; 3 Department of Crop Sciences, Institute of Agronomy, University of Natural Resources and Life Sciences Vienna, Konrad-Lorenz-Straße 24, 3430 Tulln, Austria; 4 Department of Soil Science of Temperate Ecosystems, Georg August University of Goettingen, Büsgenweg 2, 37077 Göttingen, Germany; 5 Department of Aeronautics and Astronautics, National Cheng Kung University, University Road/70101, Tainan 70101, Taiwan or; 6 Research Center for Smart Sustainable Circular Economy, Tunghai University, Taichung 407, Taiwan; 7 Department of Mechanical Engineering, National Chin-Yi University of Technology, Taichung 411, Taiwan; 8 Department of Pedotechnics, Faculty of Agriculture, Iasi University of Life Sciences, 3 Mihail Sadoveanu Alley, 700490 Iasi, Romania

**Keywords:** waste management, carbon cycle, GHG emissions, soil amendment, plant health

## Abstract

Sewage sludge (SS) has been connected to a variety of global environmental problems. Assessing the risk of various disposal techniques can be quite useful in recommending appropriate management. The preparation of sewage sludge biochar (SSB) and its impacts on soil characteristics, plant health, nutrient leaching, and greenhouse gas emissions (GHGs) are critically reviewed in this study. Comparing the features of SSB obtained at various pyrolysis temperatures revealed changes in its elemental content. Lower hydrogen/carbon ratios in SSB generated at higher pyrolysis temperatures point to the existence of more aromatic carbon molecules. Additionally, the preparation of SSB has an increased ash content, a lower yield, and a higher surface area as a result of the rise in pyrolysis temperature. The worldwide potential of SS output and CO_2_-equivalent emissions in 2050 were predicted as factors of global population and common disposal management in order to create a futuristic strategy and cope with the quantity of abundant global SS. According to estimations, the worldwide SS output and associated CO_2_-eq emissions were around 115 million tons dry solid (Mt DS) and 14,139 teragrams (Tg), respectively, in 2020. This quantity will rise to about 138 Mt DS sewage sludge and 16985 Tg CO_2_-eq emissions in 2050, a 20% increase. In this regard, developing and populous countries may support economic growth by utilizing low-cost methods for producing biochar and employing it in local agriculture. To completely comprehend the benefits and drawbacks of SSB as a soil supplement, further study on long-term field applications of SSB is required.

## 1. Introduction

Organic matter makes up about 50–70% of solid waste SS [1], which contains 33.4% protein, 6.6% lipid and 3.3% carbohydrate on an organic basis, and is highly susceptible to decomposition due to the low contents of lignin and cellulose [2]. As a result of the release of hazardous metals and organic pollutants, as well as the emission of GHGs, SS has a high potential for causing environmental deterioration [3,4]. In 2020, the volume of municipal wastewater generated annually worldwide was estimated to be 360–380 km^3^ [5]. It is estimated that more than three-quarters of this amount enters surface and groundwater without treatment [6]. Drying causes the water content to decrease, leaving around 20% of the wastewater that is solid and known as sewage sludge [7]. There is not an exact amount of global SS at the moment, and all available data are presented from different countries and in different years. The estimated rate of dry solid SS on a global scale in 2018 was 45 million tons [8]. This outcome was obtained by taking into account the two billion people who were part of municipal wastewater sanitation systems with secondary treatment facilities. Therefore, managing a considerable and steadily rising amount of SS is the top priority for both developed and emerging countries. There are now many techniques to handle SS, but none of them are without drawbacks. According to their annual budgets, technological capabilities, population sizes, and rates of development, many countries often use each of these techniques. Due to a lack of legal and financial resources, SS management has received little attention throughout a considerable portion of the world (including several nations in South America and Africa). As a result, several disposal methods, including landfilling, incineration, and dumping into the sea [9,10], have been used as the easiest common practices, causing negative effects on the environment, especially through GHG emissions into the atmosphere. Strict laws in Europe have led some EU countries (such as Germany and the Netherlands) to ban the landfilling of SS [11], while 50% of SS is managed by landfilling in the United States [12]. Around 35% of SS is used as fertilizer in Europe and the United States [1,13]. In Japan, 70% of SS is managed by incineration. More than half of the SS in South Korea is dumped into the sea [14]. Additionally, the incineration of SS in Finland produced 2307 tons of CO_2_-eq emissions [9]. The results of GHG emission studies in Greece showed that 2883 tons of CH_4_ are released from SS landfill sites annually [10].

The Sewage Sludge Directive 86/278/EEC supports the use of SS in agriculture since it is the most promising method for utilizing this waste material due to its substantial concentrations of macronutrients and organic materials. Additionally, interest in organic farming is rising as a result of the harmful effects of conventional fertilizer on the environment [15]. Some papers show that SS has a positive effect on plant yields because of its clear macronutrient content [16]. Additionally, the majority of publications [9,17] are concentrated on the harmful impacts of SS, such as the potential transfer of viruses, pesticides, heavy metals, and other contaminants. Nitrate and other contaminants may, therefore, penetrate the soil more deeply when released without being treated [18,19,20].

**Figure 1:**
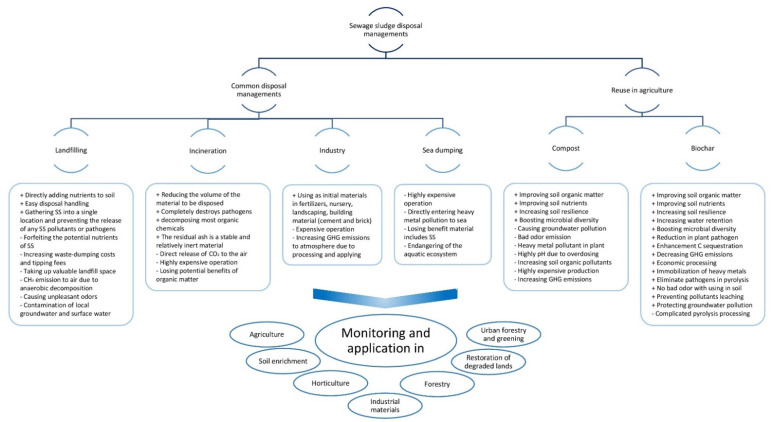
Summary of scope and content of the review focusing on environmental effects of common SS disposal managements compared with reuse in agriculture.

Various feedstock are used nowadays for the production of biochar, including crop residues, woody materials, green wastes, and animal manures [21,22]. Numerous studies have been conducted on the production of biochar from SS, its characterization, and the evaluation of its impact on soil and crop qualities. Although there are reviews on the characteristics of biochar and its use in soil [23,24,25], a special assessment on SSB is still necessary because of its enormous potential for large-scale production and the mitigation of environmental hazards. This review combines studies on the approximate volume of the world’s SS production, as well as the feasibility of using SS to support sustainable agriculture. The socioeconomic perspective of SSB production in comparison with other typical SS disposal management methods is also considered. The scope and substance of the review are summarized in Figure 1.

## 2. Importance of SS as Feedstock for Biochar Production

Sewage sludge (SS) as a huge soil C stock is a byproduct of wastewater. The presence of various pollutants, including heavy metals in SS, has irreversible destructive effects on the environment [20,26]. A significant portion of the world’s SS is produced in East Asia, Europe and North America [11]. China annually produces over 13 Mt DS year^−1^ (million tons dry solid per year) of SS [27]. In the United States, annual SS production has reached almost 8 Mt DS year^−1^ [28]. Additionally, the amount of SS produced in the European Union per year was 10 Mt DS in 2006, and 11.5 Mt DS in 2015 [29,30]. Alternatively, SS could be utilized in a wide range of manners, including land reclamation, horticulture and landscaping, industrial operations, and energy recovery [31,32]. Heavy metal contamination and nutrient surpluses in SS affect organisms in more than simply agricultural soils. They also penetrate groundwater, surface waterways, and nearby ecosystems, including protected natural areas, through leaching, run-off, and volatilization [33]. Because these places are naturally uncontaminated and are typically acclimated to low nutrient supply, this can seriously damage the structure and biodiversity of organisms [34]. As a consequence, in environmental protection processes, limiting heavy metal exportation paths and preventing their development should be a top goal. The concentration of SS organic contaminants, including as heavy metals and pathogens, may have significant implications for human food safety and plant health [35]. Therefore, there is a need to improve SS treatment solutions to address the choice towards options that guarantee safety, environment protection, economic advantages, and social sustainability. The conversion of SS into biochar through the pyrolysis process can result in multiple aspects including energy production, sustainable waste recycling, the immobilization of heavy metals and organic pollutants, C sequestration, improvements in soil quality, plant development, and mitigating GHG emissions [36].

## 3. SSB Production and Characterization

Pyrolysis and gasification have proven to be clean and cost-efficient solutions for SS treatment [37,38,39]. As a result, suitable techniques of minimizing SS waste and then GHG emissions should be established. This will enhance soil functioning while also increasing carbon sequestration [40]. In many ways, converting SS to biochar can be advantageous for environment. Some of them are: reducing the volume of sludge abandoned, reducing the cost of disposal, controlling groundwater pollutants, increasing soil carbon sequestration, and reducing GHG emissions [16,41,42]. Pyrolysis and incineration, on the other hand, are two thermal processes with various extents of efficiency. Incineration is the most studied and used thermal procedures for SS treatment right now. The circulating fluidized bed is ideal for incinerating dried SS with a high heat calorific value [40]. The main advantages of this technology are high energy efficiency, and relatively low investment compared with other technologies. This technique, however, necessitates drying as a mandatory pre-treatment [43]. Furthermore, ash is created during the incineration, which can include deposited heavy metals from the SS [44]. As a consequence, it necessitates adequate treatment in order to avoid environmental damage. Pyrolysis, on the other hand, is a highly endothermic process that necessitates 100 KJel kg^−1^ DS [45,46]. This procedure also necessitates the loss of moisture. In fact, in the incineration process, SS is burned to produce energy, but in pyrolysis, energy is used to produce biochar as the final product. This could be the basic pillar for the decreased use of pyrolysis. The immediate emission of GHGs into the environment is reduced by turning the discarded SS into biochar. By applying SSB to the soil, we can expect another reduction in GHG emissions [38,42]. This mainly occurs through the increase in carbon sequestration in soil [47]. In addition, no negative effects of SSB application on the environment have been reported [48]. Table 1 summarizes the selected basic characteristics of SS and SSB produced at different pyrolysis temperatures.

## 4. Characteristics of SSB

### 4.1. PH

**Figure 2:**
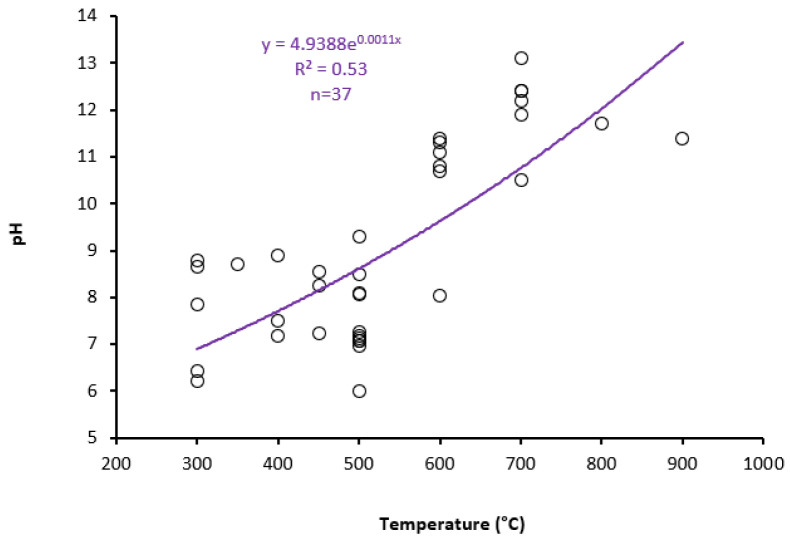
Regression relationships between pH and pyrolysis temperature.

For biochars made from sewage sludge, an increase in pH is often seen as the pyrolysis temperature rises. The pH range of the biochar obtained from sewage sludge ranges from 10.0 to 13.0 at temperatures above 700 °C [42,50,53,61]. The content of inorganic components in the biochar as a result of the separation of metal salts from the organic matrix at rising temperatures, dehydration associated with a reduction in acidic surface groups during thermal treatment, and polymerization/condensation reactions of aliphatic compounds could all contribute to this increment [62]. Additionally, there is a strong positive relationship between the pH of SSB and the pyrolysis temperature (Figure 2).

### 4.2. Elemental Composition

**Figure 3:**
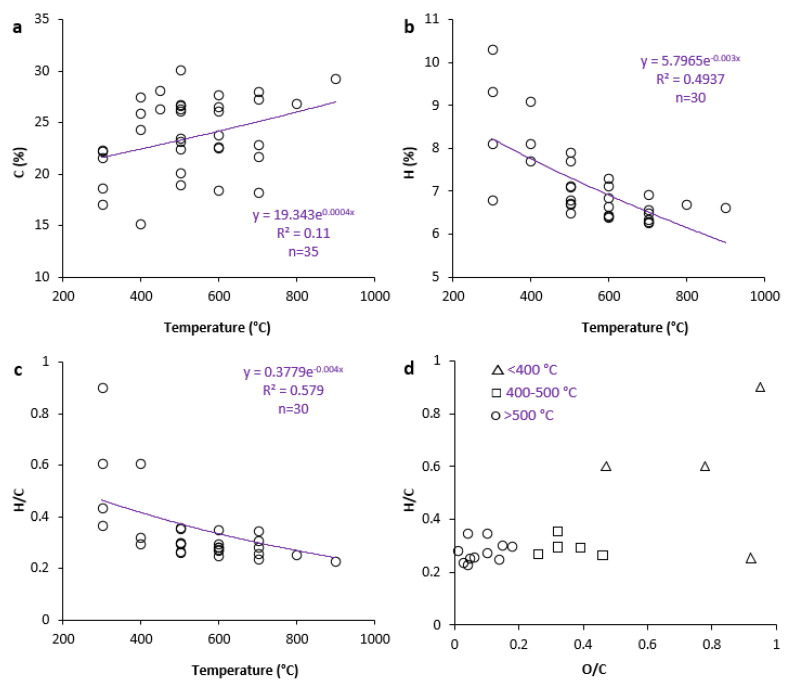
Relationship between traits for sewage sludge biochars and their pyrolysis temperature and Van Krevelen diagram for rice husk biochars produced at different pyrolysis temperatures. (**a**) C % vs. Temperature; (**b**) H % vs. Temperature; (**c**) H/C % vs. Temperature; (**d**) H/C % vs. O/C.

C, H, O, N, P, and K are the primary components of SSB. Pyrolysis temperature and the C and H contents in SSB reveal substantial positive and negative relationships, respectively (Figure 3). Raising pyrolysis temperature often causes an increase in the C content of SSB [42]. Most noteworthy, however, is that compared with SS, C in SSB is mostly found in more stable forms. The H content of SSB reduces with rising pyrolysis temperature, in contrast to C content, and in comparison to SS (Figure 3 and Table 1). H/C and O/C ratios are useful measures for estimating the degree of carbonization and biochar production from starting materials [63]. By raising the temperature of the pyrolysis process, O and H levels fall during the moisture loss and decarboxylation processes, which lowers the H/C and O/C ratios [48]. Increased H and O loss suggests the higher carbonization of SS, higher biochar hydrophobicity, more fused aromatic ring formation, and a harder C structure [64]. The aromaticity and polarity of biochars are calculated using the molar ratios of H/C and O/C [63]. The level of aromatic C components in SSB increases when these ratios decrease. The results reveal a strong opposite relationship between pyrolysis temperature and H/C ratio (Figure 3c). The lower hydrophilicity of SSB obtained at higher pyrolysis temperatures is demonstrated by a reduction in the H/C ratio [65]. High-temperature (>500 °C), synthesized SSB is likewise highly aromatic and highly carbonized [66]. Regarding the H/C ratio, it is recommended that biochar and initial material be distinguished by a maximum value of 0.7 and that soot and biochar be distinguished by a minimum value of 0.2 [64]. The ideal H/C and O/C ratios for SSB are found at pyrolysis temperatures between 350 °C and 500 °C, according to Table 1. To put it another way, this temperature range would be appropriate for the preparation of SSB. To create a Van Krevelen diagram, molar H/C and O/C ratios of SSB obtained at various pyrolysis temperatures are utilized (Figure 3d). Biochars with O/C ratios more than 0.6 and less than 0.2 have half-lives of around 100 and more than 1000 years, respectively [64]. According to Figure 3, RHBs synthesized at various pyrolysis temperatures (most often with an O/C ratio of 0.2–0.6) have a half-life of 100–1000 years.

## 5. Sewage Sludge Biochar as Soil Amendment

Pyrolytic processes with extensive applications may be used to produce biochar for a reasonable price from a range of wastes [61]. SSB is applied as a soil additive because it has a high porosity, broad surface area, high nutritional content, and an excellent water-storage capacity [25]. A portion of the pollutants from the soil is eliminated when sewage sludge is converted into biochar, and the bioavailability and mobility of heavy metals are decreased [23]. Future developments in SSB are anticipated to concentrate on the uses for which it will be used, given that the quality and effectiveness of biochar varies greatly depending on the source material and pyrolysis circumstances [24]. A crucial factor for identifying SSB’s possible application is its composition [25]. Physical and chemical characteristics of biochar can include bulk density, surface, electric conductivity, pH, cation-exchange capacity, mole ratio, concentration of different nutrients, and contaminants [37,38,39]. The application of SSB in the soil is a beneficial agricultural practice that enhances soil’s physical and chemical properties, decreases GHG emissions, improves the efficiency of using nutrients, increases crop productivity, decreases nutrient losses through leaching, changes the content and availability of nutrients, treats contaminated soils, decreases soil erosion, and improves soil structure and fertilizer efficiency [16,41,42]. The effects of SSB application on soil and plant growth are represented in Table 2.

The functional groups of the biochar used in this study were analyzed using a Fourier transform infrared (FTIR) spectrophotometer in the mid-infrared region, from 4000 cm^−1^ to 400 cm^−1^. The results of the FTIR spectra are shown in Figure 1.

## 6. Comparison between SSB and Sewage Sludge Compost (SSC) for Agricultural Purposes

Agricultural conversion attempts to advantageously utilize the organic matter and plant nutrition in biosolids, whereas landfilling and incineration constitute a one-way flow of energy and material from production to disposal [11]. Biosolids obtain the majority of their organic matter and nutrients from crops cultivated on agricultural fields [79]. Biosolids are returned to the soil by land application, where they might be used to grow new crops [80]. Composting is one of the ways of managing SS which may be widely applied in agriculture, horticulture, and forestry to restore degraded land through supplying high contents of organic matter and nutrients [1,33,79,81,82,83]. The process of composting is an aerobic method during which biodegradables are decayed to stable humic components with the participation of microorganisms [79,84]. The final compost product contains a high amount of decomposed organic matter with low amounts of heavy metals and pathogens compared with pure SS [79]. Sewage sludge compost (SSC) causes an improvement in soils and plants when combined with various additives (bulking agents) during the composting process, e.g., straw, bark, rice husk, sawdust, woodchips, and green and dry plant wastes [17,79,85]. SSC and SSB are rich with organic matter and nutrient storage, encouraging the growth of many beneficial microorganisms, and these organisms have a good ability to produce various organic acid compounds that help with nutrient availability or promoting plant growth [38,73,86,87,88]. Nevertheless, these two types of organic matter are not the same in terms of intrinsic properties and environmental impact.

### 6.1. Nutrient Status

SSC with rice husk resulted in substantial elevations in soil nitrogen and carbon, as well as soluble organic C [79]. Similarly, the addition of digested SS composted with sawdust and woodchips affected N content in spodosol and oxisol soils, while SSC inhibited mineral N production [85]. In contrast, SSB amendment is conductive to promoting carbon sequestration, enlarging soil carbon pools, and lessening the emission of GHGs [53]. SS contains various nutrients, especially potassium and phosphorous, which are potent fertilizer sources [89]. SSB could be a definitive source of P, and it contains a mix of inorganic polyphosphates, along with intrinsically attached P [90]. The SS becomes elevated in total P following carbonization [77]. This is due to the lower volatility of P during the carbonization process, which normally entails temperatures ranging from 300 to 900 °C in dry pyrolysis [91]. The enhancement of total P during carbonization, on the other hand, is associated with increased P fixing in ash compounds [92]. SSB contains one to two orders of magnitude more total P than SSC [93]. It has been widely shown that SSB is most effective for increasing the sorption capacity of soils, outperforming SSC [94]. Furthermore, SSC preparation takes longer and involves extra logistics compared with SSB, which is practically ready after the pyrolysis process [88]. Biochar is characterized by its higher content of stable organic carbon compounds compared with compost, and thus it slowly decomposes in the soil [68]; thus, it becomes more effective at improving the soil’s physiochemical properties [25].

### 6.2. Plant Growth

Compost (produced from green waste and SS from municipalities) added to sandy soil in combination with biochar and plant growth-promoting rhizobacterial inoculum resulted in higher P and K availability due to greater microbial activity [95]. Soil enrichment with SSC may improve conditions for plant growth and result in slow mineralization and the slow release of micronutrients from the compost, which are taken up by plants in very small amounts, depending on the plant and soil conditions [33,34]. In addition to nutrient content, the application of SSC may also create better soil conditions through an increase in porosity and bulk density, an improvement in moisture retention and aggregation, and an increase in soil resilience due to organic matter addition [81,86]. The positive effect of a proper dose of SSC on plants has been confirmed in various species, e.g., *Mangifera persiciforma* [86], *Phaseolus vulgaris* [96], *Rhamnus* [82], and *Brassica oleracia* [97]. SSC application (once or twice per rotation cycle) considerably increased the soil content of accessible forms of copper (Cu) and zinc (Zn), which are essential for plant function, according to a study on enriched brown podzolic soil [83]. SSC increased Zn and Mn levels in grains, whilst Fe and Ni were accumulated in plant shoots [34]. It was reported that SSC addition resulted in an increase in the leaf biomass of different trees (*Quercus acutissima, Liriodendron tulipifera*, and *Betula schmidtii*) and changed the trees’ physiological parameters, with the simultaneous accumulation of a safe level of heavy metals both in leaves and in soil [81]. A number of cereals, tubers, roots, and fibers show positive response to SSB addition in tropical, subtropical, and even temperate regions [73].

### 6.3. Heavy Metals and Pesticides

In addition to nutrients, waste may also contain harmful substances. Therefore, monitoring the quality and the dose of SSC, based on legal recommendations, is necessary prior to application thereof in the environment. Composting is a process by which environmentally harmful SS changes its properties and can be used as a soil additive, which is an important part of circularity. Composting limits the solubility and potential bioavailability of heavy metals in soils as a result of their complexation in organic matter [79,98]. However, in the context of potential contamination, e.g., by heavy metals and organic pollutants, it is important to monitor the amount and quality of SSC added to soil [99,100]. SSC and SSB, depending on their properties, may produce varying levels of bioavailable forms of potentially toxic elements (PTEs) such as Cd, Cr, and Zn when used as soil supplements [101]. With repeated applications, SS-derived PTE can contaminate the soil and accumulate in crops to levels that pose a risk to human and animal health [102,103]. In broccoli, applying SSC to the soil resulted in greater levels of Cd and Pb [104]. Swiss chard has been shown to absorb high amounts of Cu and Zn [105]. Due to its sorption mechanisms, biochar helps lower high concentrations of soluble metals such as Cd and Zn from polluted soil [25]. Heavy metals were deposited in the topsoil (0–20 cm) of barley grains following the application of SSC, according to the findings of a three-year field study on farmland soil [106]. SSC should be applied to agriculture at a low rate (150 tons per hectare), according to the authors. Likewise, it has been confirmed that fresh SSC content has an impact on the dispersion of polycyclic aromatic hydrocarbons (PAHs) in soils planted with *Festuca arundinacea*, with accumulated PAH in its tissues [99]. Although composting can effectively remove some organic contaminants from SS, the levels of some recalcitrant organic pollutants (e.g., polychorodibenzo-p-dioxins and polychorodibenzofurans (PCDD/Fs), polychlorinated biphenyl (PCBs), and perfluorinated compounds (PFCs)) sequestrated in organic fractions may even increase after organic matter decomposition [17]. In addition to the potential risk of contamination of soil and plants, SSC over-dosing can also increase pH, which may not always be suitable for all plants, as in the case of, e.g., the 45% dose of compost applied to *Rhamnus* and *Myrthus* plants [82]. From an environmental standpoint, pyrolysis is seen as a viable technology for the advantageous reuse of SS [107]. SS volume is decreased by 80% after pyrolysis, and dangerous substances such as pathogenic organisms, heavy metals, and organic and inorganic components are immobilized in biochar to prevent leaching [61]. According to reports, SSB’s heavy metals are effectively immobilized at 500 °C [66]. Ass a result, the biochar produced by pyrolyzing SS does not contain harmful pathogens and is rich in nutrients and carbon [61]. SSB has drawn the attention of most researchers for minimizing heavy metal accumulate in crops [76] and its potential benefit of nourishing soil in agriculture fields [108]. The ash in SSB can have a considerable liming impact, reducing soil acidity, Mn^2+^, Al^3+^, and other heavy metal toxicities [91]. Various researchers have shown that biochar produced through the pyrolysis method can totally eliminate microorganisms, stabilize heavy metals in SS, and diminish mineral nutrient bioavailability [38]. In terms of pesticide filtration and carbon sequestration, biochar surpasses compost treatments [94]. SSB additions often enhance pesticide adsorption in soil, according to a wide number of studies [108]. The key parameters influencing SSB’s sorption capability are its porous structure and chemical characteristics [42]. The threat of Cu, Ni, and Zn drainage from SSB-supplemented soil has been observed to be negligible [109]. Plants growing on SSC-supplemented soils acquire more PTEs [87], presumably due to compost-derived PTE’s greater leaching ability [17]. As a result, it has been recommended that SSC could be used as a soil amendment in forests but not on farmland [94]. Overall, SSB as a soil amendment appears more appropriate than SSC due to its ability to immobilize and reduce PTEs.

### 6.4. Organic Pollutants

The use of SSC has some benefits for soils, plants, and circularity, but its introduction into the soil requires strict qualitative and quantitative control according to the limits specified in the current regulations. The microbiology of the soil could be altered by adding compost to it. SSC-treated soils had higher levels of active microbial biomass than chemical fertilizer-treated soils [87]. In a tailing environment, the enrichment of microbial community diversity and an increase in the richness of *Proteobacteria* and *Ascomycota* were found in a treatment consisting of returning alfalfa green manure and SSC [33]. SS loses pathogens that are killed in the thermophilic phase of the process by composting [79]; additionally, the use of SSC may minimize plant pathogens [110]. However, on the other hand, in addition to the many benefits of SSC, there is also an increase in chemical and microbiological risks [88]. SSC enhanced the durability of *Salmonella enterica*, which invaded some plants, demonstrating the importance of safety concerns [88]. In contrast, no negative effects on the environment have been documented in the preparation of biochar from SS. In many ways, converting SS to biochar could be helpful to the environment. Some of them are: reducing the volume of sludge abandoned, reducing the cost of disposal, controlling groundwater pollutants, increasing soil carbon sequestration, and reducing GHG emissions [111].

### 6.5. Processing

In addition to the economic aspects of SSC production and transport, its use in agriculture may also be limited by the risk of contamination with heavy metals, pesticides, insecticides, different organic pollutants, hormones, pharmaceuticals, and detergents, which may be included in the food chain through soil and plants [17,79,87]. On the other hand, pyrolysis is an alternative technology that is clean and cost-efficient for treating organic wastes [46]. By turning SS to biochar, the volume of SS could well be greatly decreased while also controlling environmental contaminants [112].

## 7. Importance of SS Conversion from the Perspective of GHG Emission Concerns

### 7.1. SSB Potential to Reduce GHG Emissions

There are already a number of misconceptions about how biochar affects soil nitrogen fractions [113,114]. Biochar is also being suggested as an “electron shuttle” for transferring electrons to denitrifying bacteria [115], advancing to the final phase in the denitrification. As a consequence, N_2_O, as a greenhouse gas released mostly in agriculture, could be reduced. Other investigations and reviews have repeatedly verified this impact. The synthesis of bacterial N_2_O reductase was boosted in a study with water-saturated soils treated with biochar, resulting in a reduction in N_2_O emissions. Most of the biochar treatments, on average, decreased N_2_O emissions by 13% and 38%, respectively [114]. This was observed in a variety of investigations using various feedstocks, biochar characteristics, and soil properties. Due to the application of biochar, nitrate concentrations were found to be reduced by 12% on average [116]. Similarly, a study on calcareous soil with biochar from fir sawdust revealed that emissions of soil-produced N_2_O were reduced by 37–47%, implying that the breakdown of N_2_O to N_2_ was stimulated at the same time [117]. In fertilized soils, the reduction in N_2_O emission due to SSB application even reached 87% [78].

In addition, the application of biochar to soils has mitigation potential through decreasing the emissions (or increasing the uptake) of other key GHGs. Studies of sole biochars produced from different feedstocks have shown various ability to absorb CH_4_ and CO_2_ emissions [118,119]. It was reported that potato stem and raspberry stem biochars were more efficient in the removal of CH_4_ than wood offcut biochar and sunflower husk biochar, with lower CO_2_ emissions at the same time [118]. SSB affects soil properties, e.g., increasing pH, C, N, P, K, and water retention (Table 2), thus changing the conditions for microbial activity. Improved aeration may enhance methanotroph activity through increased O_2_ and CH_4_ diffusion and, consequently, increase CH_4_ uptake [120]. By improving CH_4_ oxidation, biochar may help to offset GHGs. The impact of biochar on GHG emissions may be influenced by its properties (e.g., pyrolysis temperature, feedstock, dose), as well as the land use and soil moisture level [57,77,119,120,121]. Adding biochar reduced CO_2_ emissions in non-saturated forest soil and improved CH_4_ absorption in saturated soils [121]. Microbial tests confirmed that the stimulation of soil CH_4_ uptake by biochar was correlated with methanotroph abundance in the soil [118]. This effect was dependent on how long the biochar stayed in the soil; nevertheless, it significantly increased CH_4_ absorption at 60% WHC five years after the usage of the highest dose (30 Mg ha^−1^). The presented results concern biochar produced from many feedstocks; however, the effect of SSB on soil GHG is still not fully recognized and requires further research. The mitigating effect of SSB was observed in a rice plantation, where its addition to the soil reduced both CH_4_ and N_2_O emissions [58]. The effect of SSB on CO_2_ emissions from oxisol was dependent on the pyrolysis temperature, and the emission rate increased after the addition of SSB produced at 300 °C and 400 °C, while it was reduced in the pyrolysis condition of 600 °C [77]. Similarly, the enrichment of luvisol with SSB resulted in increased respiration (when pyrolysis was at 400 °C), while the CO_2_ emission rate was similar to the soil without the additive when added SSB was produced at 600 °C [57]. According to reports, each tonne of dry SS during dehydration and pyrolysis operation produced around 1.5 t of CO_2_-eq emissions, and considering the final application approach of SSB, at least 0.3 t to 0.9 t of CO_2_-eq emissions were stored as stable carbon in SSB [122,123,124]. In comparison, under the same scenarios, the net C outputs from conventional SS disposal managements reached at least 2.5 t of CO_2_-eq emissions per t of dry SS [122]. Hence, turning SS into SSB provides many benefits for the C balance over the conventional disposal of SS. It has been reported that the main variables impacting C balance in the SSB soil system are the water and C contents of the SS and the use of dewatering agents [125]. This demonstrates pyrolysis’ capacity for C sequestration and offers crucial support for the management of SS.

### 7.2. Comparison between SSB and Other SS Methods for Managing GHG Emissions

#### 7.2.1. Landfilling

Landfilling is arguably the easiest approach in terms of disposal management [126]. By concentrating SS into a single site, landfilling avoids the release of any SS-borne contaminants or diseases [127,128]. During landfilling, one of the common disposal management methods, the release of GHGs is inevitable [129]. It has been proven that SS landfill is the main cause of leachate transfer to soil depth and direct CO_2_ emission into the atmosphere [130]. In landfills, organic wastes decompose anaerobically, releasing methane (CH_4_) gas, which could be discharged into the atmosphere [131]. Other gases emitted by landfills can emit offensive scents. It has been reported that landfilled SS may also cause emissions of 60.6 kg CH_4_ t^−1^ [131]. Low access to oxygen and low initial humidity play an important role in intensifying GHG emissions [67,132]. In an anaerobic environment, methanogens are a main determinant of CH_4_ synthesis. A shortage of oxygen characterized by the rapid decomposition of organic matter during the thermophilic phase (20–50 °C) near the deposited SS results in anaerobic areas [133]. However, N_2_O is the culmination of a number of reactions that include denitrification and partial nitrification during the conversion of NH_3_ to NO_2_^−^ [134]. The results of GHG emission studies in Greece showed that 2883 tons of CH_4_ are released from SS landfill sites annually [10].

#### 7.2.2. Incineration

Incineration, as a common disposal management method, plays a major role in the direct release of CO_2_ into the atmosphere [1,9,10,18]. Incineration produces CO_2_ and also other volatile contaminants into the atmosphere [135,136]. To remove fine particle matter (fly ash) and volatile impurities from flue gasses, incinerators require complex systems [137]. SS incineration, which requires an oxygen-rich condition for the combustion of organic matter, results in CO_2_ emission [129]. As a consequence, incineration is one of the costly SS removal alternatives. In Japan, 70% of SS is managed by incineration [138]. For example, the incineration of SS in Finland produced 2307 tons of CO_2_-eq emissions [9].

#### 7.2.3. Composting

In addition to the risks of SSC application to soil, an environmental problem is posed by the emission of GHGs during the composting process. The addition of magnesium chloride is recommended for reducing GHG emissions and conserving N during SS composting [139]. Composting may result in the release of about 10–15% of GHGs [140]. For example, about 26,326 kg CO_2_, 54 kg CH_4_, and 0.37 kg N_2_O t^−1^ SS can be emitted via composting [141]. CO_2_ emissions are unavoidable during composting when organic matter mineralization, temperature, and pH are considerable [142], while CH_4_ and N_2_O emissions are major GHGs. However, earlier research has shown that through organic waste composting, more than 30–40% of total organic carbon and 70–74% of initial total nitrogen are dissipated [143]. The majority of total organic carbon is dissipated in the form of CO_2_ emissions [144], whereas 10–46% of total nitrogen is released in the form of NH_3_ [145], and 0.1–10% in the form of N_2_O [146]. In addition, CH_4_ emissions are a consequence of anaerobic decomposition during composting [147]. Furthermore, it has been noted that when composting material settles and some anaerobic pockets form inside the material, between 0.01 and 0.03 percent of the initial total organic carbon is released in the form of CH_4_ [148].

#### 7.2.4. Industrial Recovery

The reuse of SS as building materials (such as bricks and cement) is a method for the industrial recovery of SS and disposal management [149]. GHG emissions from the production of bricks and cement are 36.5 and 89,015 kg CO_2_-eq t^−1^ SS, respectively [141]. Around 35% of SS is used as fertilizer in Europe and the United States [1,13]. Approximately 3269 kg CO_2_-eq t^−1^ is produced in the process of converting SS into fertilizer and using chemical raw materials such as H_2_SO_4_, NaOH, and NaClO. The application of the produced fertilizer also has the potential for CO_2_-eq emissions of as much as 31,125 kg t^−1^ of SS.

## 8. Socio-Economic Potential of SSB

Because of the advantages of using biochar, it is vital to think about the financial elements of its production and use. In practice, biochar is thought to be less expensive than other waste disposal methods [150]. It has been reported that the price of feedstock (USD 6.71–110/ton) for the production of biochar from a variety of sources, such as agricultural waste, wood residues, SS, and others, is significantly less than the cost of its production (USD 51–5668/ton), which includes labor, storage, and other costs [151]. While the expense of disposing of SS in landfills is assessed to be USD 195,000 per t per year [152]. The expected market price for produced SSB is roughly USD 246/ton, which is considerably cheaper than the cost of manufactured activated carbon (USD 1500/ton) [153]. At this cost, annual expenses are covered by the proceeds from the sale of biochar. Additionally, a significant and long-lasting invisible advantage of SSB is the avoidance of the costs associated with treating, transporting, and disposing of bio-waste, as well as any potential environmental risks [154]. On the other hand, its manufacture allows for the use of a variety of feedstocks, including SS, biowastes such as food waste [65], and fermentation residues from a farm’s biogas station [155], all of which contribute to the implementation of a circular economy. According to reports, landfilling does not require a significant financial investment, but it does produce gas emissions, odors, and the pollution of subsurface water [156]. Pyrolysis and incineration are both energy-intensive, reduce SS volume, and destroy pathogens and odors; however, pyrolysis is less polluting and results in nutrient buildup in biochar. Given the advantages of applying SSB to soil, which include increased yields and lower GHG emissions, this is an essential choice from both an environmental and agricultural standpoint. It is also worth mentioning that the environmental impact of SSB applications is influenced by the scope of study, as long-term field tests may yield better results than more frequent short-term laboratory studies, which come with their own set of expenses [150]. Drying, storage tanks, pyrolysis reactors, cyclones, condensers, and transport are all included in the expenses of SSB production phases [157]. Biochar production involves similar phases regardless of the feedstock utilized, which define stage-specific prices. Storage, drying, post-processing, and storage are usually the next steps after the substrate is delivered to the production site and before biochar is applied [158]. Because substrate availability and local variables have such a big impact on production costs, their contribution to the overall budget varies depending on the biomass and process circumstances. Overall, the current challenge is to find ways to reduce the cost of producing biochar and improve its economy at various stages, such as using arboricultural arising, reducing transportation costs by producing biochar on a smaller scale near feedstock sources and application sites, or using closed circuits, such as using the heat from a biogas combustion engine for pyrolysis [155,159,160]. Because the cost of the feedstock is such a large part of the overall cost of producing biochar, it is economically reasonable to use waste such as SS. Synthesis gas and bio-oils are among the bioenergy products of the pyrolysis process. Breaking the bonds that bind the molecules of biomass together is necessary to extract the chemical energy that is present in the biomass [161]. Synthesis gas is primarily made up of CO and H_2_, with a little amount of CO_2_ and other molecules. It burns readily and has a lower energy density than natural gas [162]. Synthesis gas can be utilized as a standalone energy source (for example, in gas turbines) or as an ingredient for synthetic natural gas, petroleum, or liquid fuel. The other bioenergy product is bio-oil, which can be used as a substitute for fuel oil or heating oil [163]. According to reports, the yields of SSB, tar, and syngas during pyrolysis conditions are roughly 56%, 26%, and 18%, respectively [164]. Fast pyrolysis, which occurs at high temperatures (500–1000 °C) and rapid heating rates (>2 C s^−1^), releases a significant amount of bio-oil (75%), biogas (13%), and biochar (12%) [165,166]. The resulting co-pyrolysis gas’ heating value was observed to be significantly higher than that of natural gas, and it also contained no measurable hazardous fumigants. To save energy and reduce potential tar and tail gas pollution, the produced tar and syngas, with heat values of approximately 17–36 MJ kg^−1^ [164] and 11–22 MJ kg^−1^ [167], respectively, were recycled as fuel alternatives for the pyrolysis process [168]. However, due to SS’s high humidity, pyrolyzing it by itself is not an energy-efficient process. Dewatering before pyrolysis could significantly lower the energy required. Due to their abundant energy supply, waste agricultural plastic films can be utilized to complement this approach. Plastic films are usually applied in soil fumigation [169]. They are mostly buried in landfills, which is costly, inefficient, and destructive for the ecosystem. If SS is co-pyrolyzed with high-energy-density substances, such as plastics with energy densities ranging from 33 to 46 MJ kg^−1^, it is predicted that the pyrolysis will become actively sustainable without the need for external energy. Co-pyrolyzation could also create extra electricity to achieve farm operations’ energy needs [167].

## 9. Approximate Prediction of Global SS and CO2-eq Emissions from it in 2050

In recent decades, population growth has played an effective role in the production of SS [18]. Annual SS productivity and population increase were shown to have a Pearson correlation coefficient of more than 0.75 (*p* < 0.01), which indicates the inevitable effect of population growth on SS production [14]. Additionally, a correlation higher than 0.8 (*p* <0.01) between population density and total GHG emissions was reported in [18]. To calculate the worldwide CO_2_-eq emissions emitted from SS, we used the world population as a basis. There are two reasons for this: (1) Despite a limited number of case studies, data on SS production capacity and disposal management from many developing and undeveloped countries have not been reported due to a lack of thorough studies. For example, a clear capacity of annual SS production is not available in populous countries such as Nigeria, Ethiopia, and Egypt in Africa, and Indonesia, Pakistan, and Bangladesh. (2) Some developed countries have not provided a new assessment of annual SS production in the last seven years [30]. CO_2_-equivalent calculations could be used to calculate the global warming potential (GWP) of three greenhouse gases (CO_2_, CH_4_, and N_2_O). It is simple to compute the total quantity of CO_2_-eq emissions produced by using the coefficients of 1, 25 and 298 CO_2_-eq emissions for CO_2_, CH_4_, and N_2_O, respectively [29]. To evaluate the current global capacity of SS in CO_2_-eq emissions and to estimate the capacity for 2050, we must first determine the total volume of SS produced globally. Then, by considering different management methods and the amount of CO_2_-eq emission in each approach, the total CO_2_-eq emissions emitted worldwide from SS can be calculated.

According to statistics, the world’s population at the end of 2020 was 7.8 billion, and this figure is predicted to rise by roughly 20% by 2050 [170,171]. Average wastewater and SS production per capita and day are predicted to be 246 L and 0.04 kg (dry solid), respectively [172,173]. Based on hsistorical data from different countries and previous studies, the contributions of disposal methods to annual SS production were assumed as follows: 26 to 70% by incineration (average 48%); 20 to 28% by landfill (average 24%); 14 to 20% by land application in agriculture, horticulture and forestry (average 17%); and 7 to 15% by other methods such as sea dumping, producing building material, and so on (average 11%) [1,16,38,45,69,174,175,176]. The potential of four routes of SS management for CO_2_-eq emission by tons CO_2_-eq t^−1^ DS (t CO_2_-eq t^−1^) were considered as follows: 223.02 t CO_2_-eq t^−1^ by incineration, 1.564 t CO_2_-eq t^−1^ by landfill, 31.125 t CO_2_-eq t^−1^ by land application, and 93.731 t CO_2_-eq t^−1^ by other management methods [142]. Then, for each continent, the proportion of the four SS management methods to CO_2_-eq emissions was computed as follows:

(1) $${\mathrm{CO}}_{2}{\;t}^{-1}\mathrm{DS}=M_{1}\left(223.02\right)+M_{2}\left(1.564\right)+M_{3}\left(31.125\right)+M_{4}\left(93.731\right)$$

where M_1_, M_2_, M_3_, and M_4_ represent the contribution percentages of incineration (48%), landfill (24%), land application (17%), and other management methods (11%), respectively.

**Figure 4:**
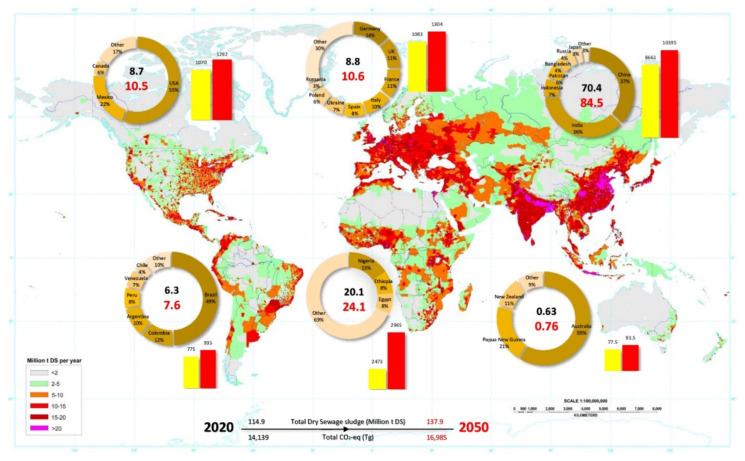
Global map of SS production and CO_2_-eq emissions from it. The inset doughnut graphs show the share of SS-producing countries by percentage from 6 continents (North and South America, Europe, Asia, Africa, and Oceania) in 2020. The inner bold numbers of the doughnut graphs indicate the total SS produced in 2020 (black), and the predicted values for 2050 (red) in million t DS. The bar graphs show CO_2_-eq emissions from different SS managements in 2020 (yellow) and the predicted values for 2050 (red) in Tg CO_2_-eq t^−1^ DS.

Figure 4 is a schematic prediction of SS production in million tons of dry solid per year (Mt DS year^−1^) of CO_2_-eq emissions in teragrams (Tg) in 2020 and 2050, according to the latest statistics of continental population distribution.

Based on population statistics [170] and the mean capacity of SS produced per capita [171], the estimation has been made that about 115 Mt DS was produced worldwide in 2020 (Figure 2). Hence, in 2050, this figure will reach 138 Mt DS, due to a 20% increase in world population [170]. Predictably, an increase in CO_2_-eq emissions is inevitable, so that the CO_2_-eq emission of 14,139 Tg in 2020 will reach 16,985 Tg (20% increase) in 2050. With 70.4 Mt DS, Asia was the world’s greatest producer of SS in 2020. This figure will increase to 84.5 Mt DS by 2050. Moreover, 73% of all Asian SS is produced by China and India, with one-third of the global population [170]. According to calculations, the total CO_2_-eq emission value in Asia was 8661 Tg in 2020, which will increase to at least 10,395 Tg in 2050.

Following Asia, Africa was the second-largest producer of SS in the world with 20.1 Mt DS and 2473 Tg CO_2_-eq emissions from it in 2020. These amounts could reach 24.1 Mt DS of SS and 1235 Tg CO_2_-eq emissions by 2050. Nigeria, Ethiopia and Egypt, the three most populous countries in Africa, produce 31% of the continent’s SS. The other African countries have an almost equal share of SS production. Europe and North America have an almost equal share in the production of SS and, consequently, CO_2_-eq emissions in 2020 and 2050. The calculated SS production and CO_2_-eq emissions for these continents in 2020 are about 8.8 Mt DS and about 1070 Tg CO_2_-eq, respectively. These numbers will increase to 10.5 Mt DS and 1300 Tg CO_2_-eq emissions in 2050. More than 50% of North American SS and CO_2_-eq emissions are produced by the USA. Based on estimates, in 2020, South America generated 6.3 Mt DS of SS and 775 Tg CO_2_-eq emissions, with 7.6 Mt DS and 935 Tg CO_2_-eq emissions forecasted for 2050. In South America, Brazil is responsible for half of this quantity. Finally, Oceania can be recognized as the producer of the lowest SS and the corresponding emissions in the world with 0.63 and 0.76 Mt DS of SS in 2020 and 2050, respectively, and about 78 and 94 Tg CO_2_-eq emissions for the same years.

Based on population calculations, the seven major populated countries of the world, i.e., China, India, the United States, Indonesia, Brazil, Japan, and Russia, have potential to produce half of the world’s SS and are responsible for CO_2_-eq emissions from it [7]. This illustrates how crucial it is to take sewage sludge management seriously in risky countries, and by adopting ecologically friendly techniques such as biochar production in densely populated regions, the amount of GHG emissions will be greatly decreased.

## 10. Conclusions

Sewage sludge management strategies should be adopted based on correct facts, such as population, local economy, and global view. Countries’ economic conditions, population growth rates, and adherence to international regulations all have a part in determining which technical approaches should be used for sewage sludge management. This review estimated that, based on current disposal managements, CO_2_-eq emissions derived from sewage sludge will rise to 24% in 2050. However, 50% of sewage sludge and related CO_2_-eq emissions are produced by the seven most populous countries in the world, and there is no doubt that governments, particularly in high-risk countries, should implement mandatory sludge-management enforcement standards. Developing countries can also contribute to economic growth by using low-cost technologies such as biochar production for agriculture in the region, as well as appropriately managing sewage sludge and minimizing GHG emissions. To achieve this purpose, environmental monitoring institutions must be mobilized, public awareness must be raised, particularly among farmers, and stringent global regulations must be enacted. Comparing this waste management method to other options already in use, such as incineration, or direct use in agriculture, the conversion of sewage sludge into biochar can be more effective. Sewage sludge biochar is of importance because it provides a variety of purposes, including isolating (sequestering) carbon in the soil, lowering GHG emissions, enhancing soil quality, and acting as a preventative measure for environment degradation. Sewage sludge biochar appears to have better soil amendment potential than sewage sludge compost because of its capacity to immobilize pathogens and heavy metals and inhibit plant uptake. Despite these findings, a careful approach necessitates long-term research into crop responsiveness, soil types, and varied environmental variables. Such research will aid a better understanding of the risks associated with employing SS-derived biochar as a soil conditioner.

## Figures and Tables

**Table 1:** Summary of selected properties of SS and SSB.

	PT (°C)	Yield (%)	Ash (%)	P	K	Ca	Mg	C	H	N	O	O/C	H/C	C/N	pH	EC (ds cm^−1^)	SSA (m^2^ g^−1^)	Ref
				(mg g^−1^)														
SS	-	-	32.8–76.14	3.4–28.4	0.54–2.05	4.04–7.4	0.57–4.1	21.6–33.2	1.24–5.2	1.32–5.74	4.4–22.5	-	-	-	5.87–7.39	2.2–4.7	1.1–7.6	[42,49,50]
SSB	300	-	-	-	-	-	-	18.6	-	3.1	-	-	-	6.00	8.65	-	-	[51]
SSB	300	-	-	-	-	-	-	22.1	-	2.9	-	-	-	7.62	7.84	-	-	[51]
SSB	500	-	-	41.1	1.61	9.71	4.32	23.4	-	3.3	-	-	-	7.09	6	-	-	[52]
SSB	300	64.3	37.4	10.4	2.25	5.33	1.35	17.1	10.3	6.2	13.4	0.78	0.60	2.76	6.2	3.3	2.88	[53]
SSB	400	56.5	49.2	11.5	2.48	5.59	1.42	15.1	9.1	4.9	7.15	0.47	0.60	3.08	7.5	0.4	7.56	[53]
SSB	500	55.3	57.4	16.6	2.75	6.01	1.68	30.1	7.7	4.3	5.91	0.20	0.26	7.00	8.1	0.5	10.8	[53]
SSB	600	53.4	63.2	18.2	2.83	6.45	2.24	26.5	7.1	3.5	5.29	0.20	0.27	7.57	10.8	0.3	12.2	[53]
SSB	700	46.6	66.6	20.1	2.91	7.8	2.56	27.2	6.9	3.1	1.63	0.06	0.25	8.77	11.9	1.3	18.3	[53]
SSB	800	42.8	68.3	19.1	3.43	8.55	2.85	26.8	6.7	2.5	1.24	0.05	0.25	10.7	11.7	0.7	19.1	[53]
SSB	900	42.2	71.2	19.5	3.35	9.14	3.19	29.2	6.6	1.2	1.16	0.04	0.23	24.3	11.4	0.4	34.2	[53]
SSB	450	-	-	-	-	-	-	-	-	1.2	-	-	-	-	8.25	1.6	-	[54]
SSB	300	-	-	42.6	2.1	8.1	8.2	21.5	9.3	5.4	-	-	0.43	3.98	-	-	4.1	[55]
SSB	400	-	-	58.8	2.4	8.4	8.4	27.5	8.1	4.4	-	-	0.29	6.25	-	-	8.7	[55]
SSB	500	-	-	59.5	2.4	8.8	8.2	26.7	7.9	3.7	-	-	0.30	7.22	-	-	10.2	[55]
SSB	600	-	-	57.6	2.8	10.4	9.3	26.1	7.3	3.4	-	-	0.28	7.68	-	-	6.3	[55]
SSB	500	-	-	-	-	-	-	-	-	1.7	-	-	-	-	8.5	5.5	-	[54]
SSB	500	-	-	-	-	-	-	26.3	-	2.6	-	-	-	10.1	7.06	0.5	-	[56]
SSB	300	-	-	-	-	-	-	22.2	8.1	3.1	-	-	0.36	7.16	8.8	-	15.6	[49]
SSB	400	-	-	-	-	-	-	24.3	7.7	3.1	-	-	0.32	7.84	8.9	-	16.3	[49]
SSB	500	-	-	-	-	-	-	20.1	7.1	2.3	-	-	0.35	8.74	9.3	-	9.43	[49]
SSB	600	-	-	-	-	-	-	22.6	6.4	1.3	-	-	0.28	17.4	10.7	-	24.7	[49]
SSB	350	-	-	-	-	-	-	-	-	-	-	-	-	-	8.72	3.04	-	[57]
SSB	300	91.1	83.2	-	-	-	-	7.53	6.78	1.3	7.13	0.95	0.90	5.79	6.43	-	5.11	[50]
SSB	500	85.7	87.9	-	-	-	-	5.63	6.48	0.7	5.21	0.93	1.15	8.04	6.96	-	15.2	[50]
SSB	700	81.2	91.9	-	-	-	-	3.96	6.29	0.4	3.36	0.85	1.59	9.90	10.5	-	13.6	[50]
SSB	450	-	-	58.2	1.78	-	-	28.1	-	3.2	-	-	-	8.78	7.22	1.73	-	[58]
SSB	450	-	-	11.1	3.01	19.9	3.59	26.2	-	1.7	-	-	-	15.4	8.54	1.1	-	[59]
SSB	500	-	-	29.2	8.01	-	-	26.1	-	2.1	-	-	-	12.4	8.06	-	-	[60]
SSB	500	54.3	73.6	54.1	9.21	8.27	0.94	18.9	6.72	2.7	4.08	0.22	0.36	7.00	7.13	-	31.8	[42]
SSB	600	51.3	77.8	59.2	10.1	9.18	1.08	18.4	6.38	2.2	1.91	0.10	0.35	8.36	11.1	-	24.1	[42]
SSB	700	48.7	79.1	63.1	10.9	9.71	1.13	18.1	6.24	1.2	0.68	0.04	0.34	15.1	12.2	-	54.1	[42]
SSB	500	50.4	68.1	58.8	14.1	6.75	1.47	23.1	6.77	3.6	4.41	0.19	0.29	6.42	7.08	-	16.3	[42]
SSB	600	46.4	70.3	64.8	15.5	6.02	1.65	23.7	6.44	3.3	2.29	0.10	0.27	7.18	11.4	-	9.01	[42]
SSB	700	43.7	74.3	68.6	16.4	7.42	1.78	22.8	6.33	2.2	0.31	0.01	0.28	10.36	12.4	-	29.9	[42]
SSB	500	54.4	69.1	54.7	12.5	1.2	1.13	22.4	6.67	3.1	4.94	0.22	0.30	7.23	7.17	-	34.2	[42]
SSB	600	51.1	70.2	53.1	13.4	1.14	1.25	22.5	6.63	2.7	4.02	0.18	0.29	8.33	11.3	-	16.2	[42]
SSB	700	49.5	72.1	56.1	13.4	1.2	1.27	21.7	6.56	2.4	3.34	0.15	0.30	9.04	12.4	-	9.21	[42]
SSB	500	45.1	64.1	96.1	1.06	1.02	3.29	26.6	7.08	3.9	4.29	0.16	0.27	6.82	7.25	-	35.7	[42]
SSB	600	43.2	63.9	92.2	1.12	1.08	2.57	27.7	6.82	3.8	3.89	0.14	0.25	7.29	8.05	-	16.2	[42]
SSB	700	40.2	68.1	95.1	1.22	1.19	2.44	27.9	6.48	2.9	0.79	0.03	0.23	9.62	13.1	-	18.1	[42]
SSB	400	-	-	-	-	-	-	25.9	-	3.6	-	-	-	7.19	7.18	0.67	-	[56]
Min	-	40.2	37.4	10.4	1.06	1.02	0.94	3.96	6.24	0.4	0.31	0.014	0.23	2.76	6	0.3	2.88	
Max	-	91.1	91.9	96.1	16.4	19.9	9.3	30.1	10.3	6.2	13.4	0.95	1.59	24.3	13.1	5.5	54.1	
Mean	-	54.4	69.4	47.8	6.0	6.7	3.1	22.4	7.2	2.9	3.9	0.3	0.41	8.7	9.1	1.5	17.6	

SS: sewage sludge; SSB: sewage sludge biochar; PT: pyrolysis temperature; C: carbon; H: hydrogen; N: nitrogen, O: oxygen, P: phosphorus, K: potassium, Ca: calcium; Mg: magnesium; EC: electrical conductivity; SSA: specific surface area.

**Table 2:** Summary of selected data on SSB effects from environmental aspects.

	Effects	References
Effects on soil properties	↑ enzyme activity	[47]
	↑ pH, N, C, ↓ bioavailable As, Cr, Co, Ni, and Pb (but not Cd, Cu, and Zn)	[58]
	↑ N, P, K	[67]
	↑ pH, EC ↓ heavy metal uptake (Pb, Zn)	[59]
	↑ N, C, P, amount of heavy metals but with low availability	[68]
	↑ P, Mg, CEC, base saturation	[69]
	↑ P, EC, pH	[54]
	↑ pH, N, C, efficiency of microbial C use, ↓ content of Pb, Cd	[56]
	↑ pH, EC, enzyme activity, the concentrations of bacteria, fungi, ammonia-oxidizing archaea, and ammonia-oxidizing bacteria, immobilization of Cr, Ni, and Cd	[70]
	↑ C, soil microbial biomass, ↓ mobility of Cd,	[60]
	↑ C, N, P, K	[38]
	↑ water retention, P sorption	[71]
	↑ field capacity, wilting point, available water in coarse- and medium-textured soils ↓ bulk density	[72]
	↑ C, N, P	[73]
Effects on plant growth	↑ shoot biomass, grain yield of rice *Oryza sativa* L.	[58,74]
	↑ growth and yield of garlic *Allium sativum* L.	[66]
	↑ growth and yield of Chinese cabbage	[75]
	↑ turf grass growth	[68]
	↑ corn yield	[59,69]
	↑ biomass of *Poa pratensis* L.	[56]
	↑ biomass and yield of wheat (*Triticum aestivum*)	[54]
	↑ biomass of Chinese cabbage	[76]
	↑ grain yield of rice; no change in grain yield of wheat	[60]
	↑ dry weight of the aboveground (stems) and belowground (roots) tomato (*Solanum lycopersicum* L.); the yield was not increased significantly	[73]
Effects on GHGs emissions	↓ or ↑ CO_2_ emission depending on pyrolysis temp.	[57,77]
	↓ N_2_O emission and ↑ CH_4_ uptake	[58]
	↓ CO_2_ and N_2_O emission in fertilized soils	[78]
	↓ CH_4_ and N_2_O emissions	[60]

↓: decrease and ↑: increase.

## Data Availability

Not applicable.

## Abbreviations

SS: sewage sludge; SSB: sewage sludge biochar; SSC: sewage sludge compost; GHGs: greenhouse gas emissions; CO_2_-eq: CO_2_-equivalent; Mt DS: million tons dry solid; Tg: Teragram.
